# Supplementary material for: Computational Drug Repurposing for Alzheimer’s Disease via Sheaf Theoretic Population-Scale Analysis of snRNA-Seq Data
Source: J Med Chem. 2026 Feb 16;69(4):4221–38. doi: 10.1021/acs.jmedchem.5c02862 (PMC12951550; doi:10.1021/acs.jmedchem.5c02862)
Supplement: Supplementary file 1 [file jm5c02862_si_001.pdf]

Supporting Information for Computational Drug Repurposing for Alzheimer's Disease via Sheaf Theoretic Population-Scale Analysis of snRNA-seq Data

Sean Cottrell[1,2], Seungmin Yoon[3], Xiaoqi Wei[1†], Alex Dickson[2,4*], and Guo-Wei Wei[1,4,5*]

[1] Department of Mathematics,
Michigan State University, East Lansing, MI 48824, USA.

[2] Department of Computational Mathematics, Science, and Engineering,
Michigan State University, East Lansing, MI 48824, USA.

[3] Department of Pharmacology and Toxicology,
Michigan State University, East Lansing, MI 48824, USA.

[4] Department of Biochemistry and Molecular Biology,
Michigan State University, East Lansing, MI 48824, USA.

[5] Department of Electrical and Computer Engineering,
Michigan State University, East Lansing, MI 48824, USA.

Abstract

Single-cell and single-nucleus RNA sequencing are used to reveal heterogeneity in cells, showing a growing potential for precision and personalized medicine. Nonetheless, sustainable drug discovery must be based on a population-level understanding of molecular mechanisms, which calls for a population-scale analysis of this data. This work introduces a sequential target-drug selection model for drug repurposing against Alzheimer's Disease (AD) targets inferred from snRNA-seq data of AD progression- involving hundreds of thousands of nuclei from multi-patient and multi-regional studies. We utilize Persistent Sheaf Laplacians (PSL) to facilitate a Protein-Protein Interaction (PPI) analysis of AD targets inferred from disease related differential gene expression (DEG). We then use machine learning models to predict repurpose-able compounds. We screen the efficacy of different small compounds and further examine their central nervous system relevant ADMET properties, resulting in a list of lead candidates for AD treatment.

keywords: Topological Data Analysis, Persistent Sheaf Laplacian, snRNAseq, Drug Discovery

*Corresponding authors. Email: alexrd@egr.msu.edu; weig@msu.edu

†Current address: Department of Mathematics, Center for Research in Scientific Computation, North Carolina State University, Raleigh, NC 27695 USA

1 Methods

1.1 Simplicial Homology

Various types of topological Laplacians have been studied from the perspective of data analysis. Persistent Sheaf Laplacian Theory enables the fusion of geometric information derived from the simplicial complex, with the non-geometric information derived from the cellular sheaf. First, we must briefly review the notion of a combinatorial Laplacian. We first define a simplex, simplicial complex, q-chain, and boundary. A 0-simplex is a vertex, a 1-simplex is an edge, a 2-simplex is a triangle, and so on. Generally, we consider a q-simplex, σ_q. A simplicial complex is then a means of approximating a topological space by gluing together the faces of simplices. More formally, a simplicial complex K is a collection of simplices such that:

1. If $\sigma_q \in K$ and σ_p is a face of σ_q then $\sigma_p \in K$.
2. The nonempty intersection of any two simplices is a face of both simplices.

A q-chain is then defined as a formal sum of q-simplices in a simplicial complex K with coefficients in $\mathbb{Z}_2$. The set of q-chains has a basis in the set of q-simplices in K, and this set forms a finitely generated free Abelian group $C_q(K)$. We then define the boundary operator as a homomorphism relating the Chain groups, $\partial_q : C_q(K) \to C_{q-1}(K)$. The boundary operator is defined as:

$$\partial_q \sigma_q = \sum_{i=0}^{q} (-1)^i \sigma_{q-1}. \tag{1}$$

where σ_{q-1} is a $q-1$ simplex. The sequence of chain groups connected by this homomorphism is then a Chain Complex:

$$... \xrightarrow{\partial_{q+1}} C_q(K) \xrightarrow{\partial_q} C_{q-1}(K) \xrightarrow{\partial_{q-1}} ....$$

It is well known that the boundary operator and the Chain Complex associated with a simplicial complex gives the number of q-dimensional holes in that topological space. Specifically, the qth Homology Group is defined as $H_q = \ker\partial_q / \mathrm{Im}\partial_q$. This is also known as the qth Betti Number, β_q. The matrix representation of the qth boundary operator with respect to the standard basis in $C_q(K)$ and $C_{q-1}(K)$ is given as $\mathcal{B}_q$. We can equip all chain groups with the canonical inner product and define the adjoint operator.

$$\partial_q^* : C_{q-1}(K) \to C_q(K), \tag{2}$$

and the transpose of $\mathcal{B}_q$, denoted $\mathcal{B}_q^T$, is the matrix representation of ∂_q^* with respect to the same basis. We can now define the q-combinatorial Laplacian matrix as:

$$\mathcal{L}_q := \mathcal{B}_{q+1}\mathcal{B}_{q+1}^T + \mathcal{B}_q^T \mathcal{B}_q. \tag{3}$$

The harmonic spectrum of the q-combinatorial Laplacian matrix reveals the dimension of the qth Homology group, or the number of q-dimensional holes in our simplicial complex. The non-harmonic spectrum then reveals further homotopic shape information.[6] Intuitively, β_0 reveals the number of connected components in K, β_1 reveals the number of loops in K, and β_2 reveals the number of 2D voids in K.

1.2 Cellular Sheaves on a Labeled Simplicial Complex

A cellular sheaf on a simplicial complex X consists of the following:

1. A simplicial complex X, where the face relation that σ_p is a face of σ_q is denoted by $\sigma_p \leq \sigma_q$
2. An assignment to each simplex σ of X a finite dimensional vector space $\mathcal{V}(\sigma)$ and to each face relation $\sigma_p \leq \sigma_q$ a linear morphism of vector spaces denoted $\mathcal{V}_{\sigma_p \leq \sigma_q} : \mathcal{V}(\sigma_p) \to \mathcal{V}(\sigma_q)$ satisfying $\sigma_r \leq \sigma_p \leq \sigma_q \implies \mathcal{V}_{\sigma_r \leq \sigma_q} = \mathcal{V}_{\sigma_r \leq \sigma_p} \circ \mathcal{V}_{\sigma_p \leq \sigma_q}$ and $\mathcal{V}_{\sigma_p \leq \sigma_p}$ is the identity map.

The vector space $\mathcal{V}(\sigma)$ is the stalk of $\mathcal{V}$ over σ and the linear morphism $\mathcal{V}_{\sigma_p \leq \sigma_q}$ is the restriction map of the face relation. A global section s of $\mathcal{V}$ is an assignment to each simplex σ an element $s_\sigma \in \mathcal{V}(\sigma)$ such that $\mathcal{V}_{\sigma_p \leq \sigma_q}(s_{\sigma_p}) = s_{\sigma_q}$ for any face relation $\sigma_p \leq \sigma_q$.

Suppose then that we have a graph, or a one dimensional simplicial complex X where each vertex v_i is associated with a quantity $q_i \in \mathbb{R}$. Denote the edge connecting v_i and v_j as e_{ij}. We can define a sheaf $\mathcal{V}$ on K such that each stalk is $\mathbb{R}$, and for the face relation $v_i \leq e_{ij}$, the morphism $\mathcal{V}_{v_i \leq e_{ij}}$ is the multiplication by q_j/r_{ij} where r_{ij} is the length of e_{ij}. The assignment $q_i \to v_i$ and $q_i q_j / r_{ij} \to e_{ij}$ is a global section, since $\mathcal{V}_{v_i \leq e_{ij}}(q_i) = \mathcal{V}_{v_j \leq e_{ij}}(q_j) = q_i q_j / r_{ij}$.

This can then be generalized to higher order simplicial complexes. Suppose we also have a nowhere zero function $F : X \to \mathbb{R}$. We can define a sheaf where each stalk is $\mathbb{R}$ and for the face relation $[v_0, v_1, ..., v_n] \leq [v_0, v_1, .., v_n, v_{n+1}, ..., v_m]$, the linear morphism $\mathcal{V}([v_0, v_1, ..., v_n] \leq [v_0, v_1, .., v_n, v_{n+1}, ..., v_m])$ is the scalar multiplication by:

$$\frac{F([v_0,v_1,...,v_n])q_{n+1}\cdots q_m}{F([v_0,v_1,...,v_n,v_{n+1},...,v_m])}$$

1.3 Persistent Sheaf Laplacian

The Sheaf Laplacian is a combinatorial Laplacian constructed from the sheaf cochain complexes. The sheaf cochain complex of $\mathcal{V}$ can be defined as follows. Let the q-th cochain group $C_q(X, \mathcal{V})$ be the direct sum of $\mathcal{V}(\sigma)$ over all q-simplices. To define the coboundary map d, we need a signed incidence relation, which is an assignment to every face relation $\sigma_p \leq \sigma_q$ an integer $[\sigma_p : \sigma_q]$ satisfying the following:

1. If dim σ_q - dim $\sigma_p > 1$, then $[\sigma_p : \sigma_q] = 0$
2. If $\sigma_r \leq \sigma_q$ and dim σ_q - dim $\sigma_r = 2$, then $\sum_{\sigma_p} [\sigma_r : \sigma_p][\sigma_p : \sigma_q] = 0$

If a signed incidence relation is given, we then define the coboundary map $d^q : C_q(X; \mathcal{V}) \to C_{q+1}(X; \mathcal{V})$ by

$$\partial^q|_{\mathcal{V}(\sigma_p)} = \sum_{\sigma_p \leq \sigma_q} [\sigma_p : \sigma_q] \mathcal{V}_{\sigma_p \leq \sigma_q}$$

Which induces a sheaf cochain complex

$$0 \to C_0(X, \mathcal{V}) \to C_1(X, \mathcal{V}) \to C_2(X, \mathcal{V}) \to ...$$

The (q+1)-th sheaf cohomology group $H_q(X, \mathcal{V})$ is defined by ker ∂^{q+1}/im ∂^q. Persistent sheaf cohomology is known to many researchers. Suppose that $f : X \to Y$ is a simplicial map and that $\mathcal{V}$ is a cellular sheaf on Y. The pullback sheaf $f * \mathcal{V}$ on X is given by

$$(f * \mathcal{V})(\sigma) = \mathcal{V}(f(\sigma))$$

and for the face relation $\sigma_p \leq \sigma_q$ of X:

$$(f * \mathcal{V})_{\sigma_p \leq \sigma_q} = \mathcal{V}_{f(\sigma_p) \leq f(\sigma_q)}$$

Given two oriented simplicial complexes X, Y, if $X \subset Y$ and the orientation of X is identical to Y, let sheaf $\mathscr{F}$ on K be the pullback of the sheaf $\mathscr{G}$ on Y,then we have the following commutative diagram:

$$\begin{array}{ccccccccc}
\cdots & \xrightarrow{\partial} & C^{q-1}(X;\mathscr{F}) & \xrightarrow{\partial} & C^q(X;\mathscr{F}) & \xrightarrow{\partial} & C^{q+1}(X;\mathscr{F}) & \xrightarrow{\partial} & \cdots \\
 & & \pi\uparrow & & \pi\uparrow & & \pi\uparrow & & \\
\cdots & \xrightarrow{\partial} & C^{q-1}(Y;\mathscr{G}) & \xrightarrow{\partial} & C^q(Y;\mathscr{G}) & \xrightarrow{\partial} & C^{q+1}(Y;\mathscr{G}) & \xrightarrow{\partial} & \cdots
\end{array}$$

where $\pi : C^q(Y, \mathscr{G}) \to C^q(X, \mathscr{F})$ is a projection map. Since π is a cochain map, it induces a map $\pi^{\cdot}$ between sheaf cohomology groups of $\mathscr{F}$ and $\mathscr{G}$, and the q-th persistent sheaf homology group is defined by:

$$\pi^{\cdot}(H^q(Y, \mathscr{G})$$

whose dimension is the q-th persistent sheaf Betti number. To extend the notion of the persistent Laplacian to cellular sheaves, we must dualize the above diagram. Note that the adjoint of π is the inclusion map $\iota : C^q(X, \mathscr{F}) \to C^q(Y, \mathscr{G})$.

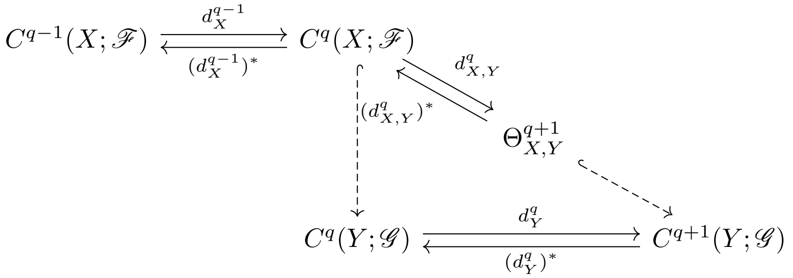

where $\Theta^{q+1}_{X,Y} = \{x \in C^{q+1}(Y, \mathscr{G}) : (\partial^q_Y)^*(x) \in C^q(X, \mathscr{F})$ and $\partial^q_{X,Y}$ is the adjoint of $\pi(\partial^q_Y)^*|_{\Theta^{q+1}_{X,Y}} : \Theta^{q+1}_{X,Y} \to C^q(X, \mathscr{F})$. We then define the q-th persistent sheaf Laplacian by:

$$\Delta^q_{X,Y} = (\partial^q_{X,Y})^* \partial^q_{X,Y} + \partial^q_X (\partial^q_X)^*$$

The nullity of the q-th persistent sheaf Laplacian is then equal to the q-th persistent sheaf Betti number. The matrix representation of the persistent sheaf Laplacian is constructed similarly to the combinatorial Laplacian.

1.4 Persistent Sheaf Laplacian Enabled Topological Perturbations

1.4.1 Protein-Protein Interaction Complexes

A PPI network is an undirected graph where each gene is represented as a node and there exists a pairwise connection between two genes if they are either experimentally known to interact or exhibit significant co-expression. In terms of their biological significance, co-expressed genes are understood to be controlled by the same transcriptional regulatory program, or they may be functionally related or members of the same pathway or protein complex.

Given the PPI network as a weighted undirected graph $G = (V, E, w)$, the clique complex of G is a simplicial complex whose simplices are all the cliques of G. A clique of G is a subset of nodes where every single node is directly connected to every other node within that subset. In this sense, we are constructing, say, 2-simplices (triangles) from the sets of three mutually connected nodes, and so on. Considering computational cost as well as conceptual simplicity, we add simplices only up to 2 dimensions, so that we may consider the L_0 and L_1 Persistent Sheaf Laplacians.

1.4.2 Topological Perturbations and Biomarker Discovery

Given our PPI clique complex, in which the genes make up the set of 0-simplexes, the STRING PPI confidence scores facilitate the construction of an inverse rips filtration.[5] We wish to now identify the topologically significant genes via topological perturbations over each scale of the filtration. We hypothesize that the genes which are most topologically significant with respect to a dysregulation scaled sheaf would correspond well to significant biomarkers of disease progression. Topological significance is calculated according to the disparity between feature vectors obtained from the original complex and a perturbed complex where the respective gene has been removed.

Specifically, for a clique complex K and a perturbed clique complex $\hat{K}$, we obtain the sets of subcomplexes induced by filtration: $\{K_0, K_1, ..., K_p\}$ and $\{\hat{K}_0, \hat{K}_1, ..., \hat{K}_p\}$. For each step of the filtration we compute the L_0 and L_1 Persistent Sheaf Laplacians. The spectra of these Laplacians encode the topological and geometric structure of our network with respect to a logFC labeling. We remove a specific gene from

the network to construct the perturbed complex. For convenience, we calculate summary statistics of the spectra: {Min, Mean, Max, Standard Deviation, Sum, Number of Zeros}, giving us a feature vector for each subcomplex. Having obtained a feature vector for K_i- call it f_i, and $\hat{K}_i$- call it $\hat{f}_i$, we calculate the Wasserstein distance between the two: $\text{dist}(f_i, \hat{f}_i)$. The larger the distance, the more topologically significant we consider that gene to be at that scale. For robustness, we then consider the set of genes that rank among the top 25 most significant out of 200 over all scales of the filtration, giving us our final set of inferred biomarkers.

1.5 Machine Learning Based Drug Discovery

Given our list of inferred biomarkers, we can cross reference with the KEGG or GO Pathway database to obtain the list of pathways regulated by these genes. Furthermore, we can conduct a review of the scientific literature to better understand the role of these genes and pathways in regulating the genesis and progression of Alzheimer's Disease. With that information at hand, we identify significant molecular targets that would be potentially suitable for treatment by repurposed drugs available in DrugBank.

1.5.1 Molecular Fingerprints

We first obtain binding affinity training data for these targets from ChEMBL. These datasets are comprised of SMILES strings for the molecular compounds, each paired with some bioactivity label, specifically IC_{50} for the inhibitors and EC_{50} for the agonists. To adapt these experimental labels into binding affinities (BAs) suitable for our models, we employed the conversion formula: BA $= 1.3633 \times$ log10Ki(kcal/mol). IC_{50} labels were subsequently estimated to Ki values based on the relationship Ki $= IC_{50}/2$, in alignment with recommendations by Kalliokoski.[2] The EC_{50} labels, meanwhile, were used to directly infer Ki values given that $EC_{50} =$ Ki can be derived from mathematical manipulations. In addition, we retrieved small molecule drugs, categorized under either approved, investigational, or experimental status from the DrugBank database (version 5.1.12). To ensure consistency, all SMILES strings were canonicalized by the RDKit toolkit.

In our binding affinity analysis, we employed two fingerprinting methodologies to delineate molecular structures in a format suitable for machine learning input. Specifically, we utilized a pretrained Bidirectional Transformer, which was pretained in a self-supervised manner using the ChEMBL27 dataset by Chen and colleagues.[1] The Biderectional Transformer was then used to transduce canonical SMILES notations into 512-dimensional latent vectors, called Bidirectional Transformer-based Fingerprints(BT-FPs). The self-supervised learning (SSL) technique in Chen et al. involved masking the SMILES strings and pairing the masked inputs with their original counterparts. The Biderectional Transformer Encoder was then used to predict the masked sections. This model sourced training data from a combination of the ChEMBL, PubChem, and ZINC databases.[3] For our purposes, we selected the model trained solely on ChEMBL samples, with no additional fine tuning.

For robustness, we also utilized a two dimensional structure based molecular fingerprinting technique called Extended-connectivity fingerprints (ECFPs).[4] ECFPs are a type of topological fingerprints for molecular characterization. The ECFP algorithm is based on a variant of the Morgan algorithm that assigns numeric identifiers to each atom through an iterative process. We used the RDKit library to generate these fingerprints, which requires a radius parameter that determines the number of iterations the algorithm should perform. In our implementation, we set the ECFP radius to 2 and the length of the ECFP fingerprint to 2048.

1.5.2 Machine Learning Based Drug Repurposing and ADMET Analysis

With suitable inputs obtained in the form of BT-FPs and ECFPs, we trained two Gradient Boosting Decision Tree regressors on the bioactivity labeled ChEMBL data, and then tested them on thousands of small compounds obtained from DrugBank. The models were finetuned using 10 fold cross validation on a 70/30 train-test split of the ChEMBL data, and after parameter tuning were refit to the entire dataset. We averaged the predicted binding affinities between the two models to obtain our final prediction. Ultimately, drugs with a predicted equilibrium dissociation / inhibitor constant (Kd / Ki) of less than 180nm (i.e. less than -9.2 kcal / mol) were deemed to be effective binders to their respective molecular target.

To align our predictions with the specific requirements of drug repurposing for AD, we applied an additional layer of filtering to the initially screened DrugBank small molecules, focusing on key CNS related physiological properties predicted by ADMETlab 3.0.[7] As a preliminary step, we excluded mixtures or ionic compounds, identified by the presence of multiple unconnected molecular entities in their SMILES representation. We then screened for compounds with a predicted BBB penetration probability greater than 70%, while excluding those with a Pgp inhibitor or substrate probability exceeding 50%.

Given the CNS involvement in Alzheimer's pathology, BBB permeability is a critical requirement for therapeutic efficacy. At the same time, compounds predicted to be either P-glycoprotein substrates or inhibitors were excluded as substrates are actively transported out of the brain, reducing central availability, and inhibitors may interfere with endogenous efflux regulation, potentially leading to safety issues or altered pharmacokinetics of co-administered drugs. We retained only those molecules that satisfied both the CNS-oriented ADMET criteria and our binding affinity thresholds: –9.2 kcal/mol for CXCR4 and ERBB4, and a slightly relaxed –8.5 kcal/mol for EGLN and PPARG due to the lack of strong candidates under the stricter threshold.

References

[1] Dong Chen, Jiaxin Zheng, Guo-Wei Wei, and Feng Pan. Extracting predictive representations from hundreds of millions of molecules. *The Journal of Physical Chemistry Letters*, 12(44):10793–10801, 2021.

[2] Tuomo Kalliokoski, Christian Kramer, Anna Vulpetti, and Peter Gedeck. Comparability of mixed ic_{50} data—a statistical analysis. *PLOS ONE*, 8(4):e61007, 2013.

[3] David Méndez, Anna Gaulton, A. Patricia Bento, John Chambers, Marc De Veij, Elisbet R. Félix, María P. Magariños, Juan F. Mosquera, Prudence Mutowo, Michal Nowotka, Carlos Gordillo-Merchán, Fiona M. Hunter, Leire Junco, Grace Mugumbate, Miriam Rodriguez-Lopez, Jan A. van Santen, Julio Perea, Anne Rives, William P. Walters, Alex J. Williams, Bissan Al-Lazikani, David Michalovich, John P. Overington, and Henning Hermjakob. Chembl: Towards direct deposition of bioassay data. *Nucleic Acids Research*, 47(D1):D1102–D1109, 2019. Accessed: 2025-05-29.

[4] David Rogers and Mathew Hahn. Extended-connectivity fingerprints. *Journal of Chemical Information and Modeling*, 50(5):742–754, 2010.

[5] Damian Szklarczyk, Annika L. Gable, Konstantinos C. Nastou, David Lyon, Rebecca Kirsch, Sampo Pyysalo, Nadezhda T. Doncheva, Maxime Legeay, Tan S. Fang, Henning Redestig, Leire Pernas, Christian von Mering, and Lars J. Jensen. STRING v12: Fast, scalable protein–protein interaction networks, integrative analysis and functional characterization of user data. *Nucleic Acids Research*, 51(D1):D638–D646, 2023.

[6] Rui Wang, Duc Duy Nguyen, and Guo-Wei Wei. Persistent spectral graph. *International journal for numerical methods in biomedical engineering*, 36(9):e3376, 2020.

[7] Guangrui Xiong, Zhixiang Yang, Jianqiang Yi, Lirong He, Liang Yu, Xiaohua Zhuang, Yu Chen, Guoqiang Hao, Xinyun Zheng, Bingjie Liu, Qi Wang, and Luhua Lai. Admetlab 3.0: An interactive platform for accurate and comprehensive predictions of admet properties of chemicals. *Nucleic Acids Research*, 51(W1):W83–W89, 2023.
